# Supplementary material for: Asialoglycoprotein receptor 1 is a novel PCSK9-independent ligand of liver LDLR cleaved by furin
Source: J Biol Chem. 2021 Sep 8;297(4):101177. doi: 10.1016/j.jbc.2021.101177 (PMC8479480; doi:10.1016/j.jbc.2021.101177)
Supplement: Supplemental Figure S1 and Table S1 [file mmc1.pdf]

Supplementary Table S1. Antibodies used for Western blotting, immunofluorescent staining, immunohistochemical staining and immunoprecipitation

Antibody	Catalog No; Source	Application	Dilution
**human LDLR**	AF2148; R&D	WB	1:1,000
**mouse LDLR**	AF2255; R&D	WB	1:1,000
**ASGR1**	11739-1-AP; Proteintech	WB	1:1,000
**α-Tubulin**	11224-1-AP; Proteintech	WB	1:5,000
**μ-Actin**	A2066; Sigma	WB	1:5,000
**Insulin receptor β**	ENZ-ABS710; Enzo	WB	1:1,000
**CD36**	Ab36977; Abcam	WB	1:1,000
**human PCSK9**	(1)	WB	1:3,000
**Flag-HRP**	A8592; Sigma	WB	1:5,000
**HA-HRP**	H6533; Sigma	WB	1:4,000
**V5-HRP**	V2260; Sigma	WB	1:10,000
**Rabbit TrueBlot®: Anti-Rabbit IgG HRP**	18-8816-31; Rockland Inc.	WB	1:2,000
**human LDLR**	AF2148; R&D	IF	1:200
**ASGR1**	11739-1-AP; Proteintech	IF	1:200
**mouse LDLR**	AF2255; R&D	IHC	1:150
**ASGR1**	11739-1-AP; Proteintech	IHC	1:300
**Goat anti rabbit IgG Alexa Fluor 488**	A27034; Invitrogen	IHC/IF	1:150/1:500
**Donkey anti goat IgG Alexa Fluor 488**	A-11055; Invitrogen	IHC/IF	1:150/1:500
**Flag M2**	F1804; Sigma	IP/IF	n/a /1:250
**V5**	R960-25; Invitrogen	IP	n/a
**ASGR1**	11739-1-AP; Proteintech	IP	n/a

WB, Western blot; IF, immunofluorescence; IHC, immunohistochemistry

1. Nassoury, N., Blasiole, D. A., Tebon, O. A., Benjannet, S., Hamelin, J., Poupon, V., McPherson, P. S., Attie, A. D., Prat, A., and Seidah, N. G. (2007) The Cellular Trafficking of the Secretory Proprotein Convertase PCSK9 and Its Dependence on the LDLR. *Traffic.* **8**, 718-732

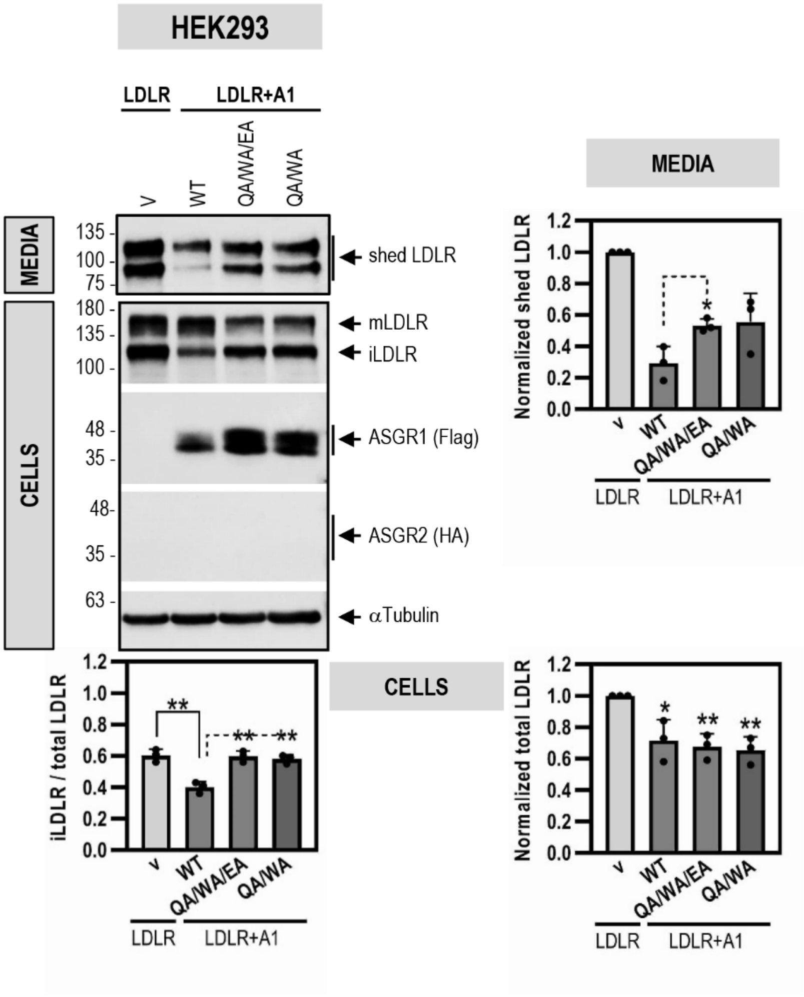

Supplementary Figure S1: The ASGR1-mediated degradation of LDLR through its lectin-binding domain is independent of ASGR2 when co-expressed in HEK293 cells

HEK293 cells were co-transfected with V5-tagged LDLR and either Flag-tagged ASGR1 (A1), WT or its carbohydrate-binding mutants Q240A/W244A/E253A (QA/WA/EA) or Q240A/W244A (QA/WA) or control empty vector (V). WB analyses of total cellular LDLR (mature mLDLR, and immature iLDLR) and ASGR1 (Flag-HRP) and of shed LDLR in the media are shown. The empty vector control (V) is the same as the one used for Figs. 7 and 9C. This is justified since the above control data are derived from the same experiment testing the effects on LDLR of ASGR2 alone (Fig. 7), and of ASGR1 in the presence (Figs. 7, 9C), or absence of ASGR2 (Fig. 7 and present Supplementary Figure S1). Quantifications are averages ± SD of three independent experiments. * $p<0.1$; ** $p<0.01$ (t-test) are relative to the control vector condition.
